# The adoption of cryptocurrency as a disruptive force: Deep learning-based dual stage structural equation modelling and artificial neural network analysis

**DOI:** 10.1371/journal.pone.0247582

**Published:** 2021-03-08

**Authors:** Ghazanfar Ali Abbasi, Lee Yin Tiew, Jinquan Tang, Yen-Nee Goh, Ramayah Thurasamy

**Affiliations:** 1 Graduate School of Business, Universiti Sains Malaysia, USM, Penang, Malaysia; 2 Newhuadu Business School, Minjiang University, Fuzhou, Fujian, China; 3 School of Management, Universiti Sains Malaysia, USM, Penang, Malaysia; Univerza v Mariboru, SLOVENIA

## Abstract

In recent years, the growth of cryptocurrency has undergone an enormous increase in cryptocurrency markets all around the world. Sadly, only insignificant heed has been paid to the unveiling of determinants of cryptocurrency adoption globally, particularly in emerging markets like Malaysia. The purpose of the study is to examine whether the application of deep learning-based dual-stage Partial Least Square-Structural Equation Modelling (PLS-SEM) & Artificial Neural Network (ANN) analysis enable better in-depth research results as compared to single-step PLS-SEM approach and to excavate factors which can predict behavioural intention to adopt cryptocurrency. The Unified Theory of Acceptance and Use of Technology 2 (UTAUT2) model were extended with the inclusion of trust and personnel innovativeness. The model was further validated by introducing a new path model compared to the original UTAUT2 model and the moderating role of personal innovativeness between performance expectancy and price value, with a sample of 314 respondents. Contrary to previous technology adoption studies that used PLS-SEM & ANN as single-stage analysis, this study further enhanced the analysis by applying a deep learning-based dual-stage PLS-SEM and ANN method. The application of deep learning-based dual-stage PLS-SEM & ANN analysis is a novel methodological approach, detecting both linear and non-linear associations among constructs. At the same time, it is regarded as a superior statistical approach as compared to traditional hybrid shallow SEM & ANN single-stage analysis. Also, sensitivity analysis provides normalised importance using multi-layer perceptron with the feed-forward-back-propagation algorithm. Furthermore, the deep learning-based dual-stage PLS-SEM & ANN revealed that trust proved to be the strongest predictor in driving user intention. The introduction of this new methodology and the theoretical contribution opens the vistas of the extant body of knowledge in technology-adoption related literature. This study also provides theoretical, practical and methodological contributions.

## Introduction

The world in the last decade has seen several technological advancements such as electronic commerce, digital payments and the internet of things (IoT). Reflections of these developments can be seen in the ways people interact and the way they exchange money [1]. Owing to the constant trend of change in the last two decades, shifting from paper to virtual currency, a new form of currency, i.e. cryptocurrency, has taken birth [2]. Mazambani and Mutambara [3] referred to cryptocurrency as a digital currency that relies on advanced encryption techniques to perform a range of financial transactions. Cryptocurrency operations are based on blockchain with a goal to provide security, transparency and anonymity [4, 5]. Furthermore, the use of cryptocurrency like bitcoins, enables users to move out of the scope of conventional retail payment methods owing to two key reasons: anonymity and non-dependence on central entities such as financial institutions [6]. Reduction in transaction time and significantly lower transactional costs are some of the few benefits that cryptocurrency can bring to potential users.

Also, the upsurge of cryptocurrency in recent years has contributed to a massive increase in the cryptocurrency market size [7]. According to Arias-Oliva et al. [8], there are more than 2,000 cryptocurrency traders in the market worldwide. Hilleman and Rauchs [9] narrated that users who actively use cryptocurrency range from 2.8 to 5.8 million worldwide. The majority of cryptocurrency users (61%) are from North America and Europe, whereas only 20% of users are actively using cryptocurrency in the Asia-Pacific region [10]. Researchers have also posited that there are also some multinational companies which accept cryptocurrency like bitcoins such as Expedia, AliExpress [11], Wikipedia, Dell and Microsoft [12]. A rise in adoption of cryptocurrency reflects a positive aspect of demand [13]. There is no doubt that the adoption of cryptocurrency is more progressive in the developed world than emerging markets, where regulators and policymakers are still hesitant to adopt it [14].

Moreover, there is glaring evidence that the extensive use of digital currencies has the potential to transform economies, particularly in the developing world [15, 16]. Similarly, Polasik et al. [12] further stated that the growth in cryptocurrency would also leave its positive impact on the e-commerce market. Miraz and Ali [17] narrated that the future of cryptocurrency looks secured, considering the worldwide rise in popularity regarding the Internet of Things (IoT). However, the fullest potential of cryptocurrency can only be attained when it has been extensively adopted [18]. Wealth of academic studies have averred that the extensive use of cryptocurrency can democratise and alter economies, particularly economies of developing nations [16]. As online traders have started accepting cryptocurrency as the medium of exchange, such as AliExpress, Microsoft and many more, its usage in consumers is still very scarce [5]. Therefore, an in-depth understanding of factors which can stimulate the adoption of cryptocurrency is pivotal. The plethora of businesses investing in cryptocurrency is an echo of positive projections of demand [13]. However, without knowing the intention to adopt, all these investments from businesses could be useless [3]. In the context of Malaysia, even though the Malaysian government has started to regulate cryptocurrency officially [19], the consumer adoption of this novel technology remains low and needs further investigation [5].

Therefore, owing to the scarcity of knowledge regarding cryptocurrency adoption, several researchers have asked for further investigation [3, 5]. In Malaysia, apart from Al-Amri et al. [5], a study which conducted a systematic literature review on cryptocurrency and highlighted the need to conduct more empirical studies and explore more about cryptocurrency; studies focusing on the individual intention to adopt cryptocurrency in Malaysia are almost non-exist. Moreover, most of the extant literature worldwide also neglects end-user adoption factors, i.e. individual user level [20, 21] as the development of cryptocurrencies may be contingent on elucidating consumer behaviour and the capability to predict the drivers which can stimulate the adoption process. Thus, it indicates adoption of cryptocurrency globally is still an evolving subject and scholars are investigating consumer intention as a proxy to their actual behaviour [15].

Furthermore, unlike a previous research using single stage structural equational modelling (SEM) [22–24], or logistics regression [25] which could only detect linear and compensatory associations among variables; it is not considered acceptable to forecast the intricacies affecting multi-faceted decision-making procedures. Consequently, to exterminate such limitations, prior studies [26, 27] have employed machine learning techniques such as artificial neural networks (ANN) as second stage of investigations entailing a single hidden layer. Moreover, studies applying SEM & ANN approaches with single hidden layers are not uncommon in numerous technology adoption studies, e.g. adoption of hybrid vehicles [28], ERP acceptance [29], wearable heath care technology acceptance [26], cryptocurrency adoption [29], m-health apps adoption [30], predicting m-government security [31]. In fact, the application of SEM & ANN in the domain of technology adoption is considered very useful [27]. Huang and Stokes [32] posited that second stage ANN analysis with single hidden layer is a shallow type of ANN. However, it is worthy to mention that all of the above-mentioned technology adoption studies have applied shallow SEM & ANN approaches, therefore, it was recommended to use deep neural network architecture based dual stage PLS-SEM & ANN approach has been recommended for better results [33, 34]. The application of deep neural network architecture with two or more hidden layers will enhance the precision of non-linear associations in the model owing to its deep learning capability [33]. Similarly, deep learning is termed as a new arena related to machine learning [34].

Thus, in an emerging country like Malaysia, responding to the call for further empirical researches and improving the existing technology adoption literature exclusively on already empirically developed cryptocurrency theoretical models, i.e. based on shallow ANN approach [29]; this study fulfils the research gap and attains both objectives of the study (a- to examine whether application of deep learning based dual stage PLS-SEM & ANN analysis enables better in-depth research results as compared to PLS-SEM approach; b- to excavate the factors which can predict behavioural intention to adopt cryptocurrency). However, this study does not try to compare the results of deep neural networks results with shallow ANN approach. The aim of the study is to compare the findings generated, by applying deep neural networks with PLS-SEM findings. Thus, following the suggestions of [32–34], this study also used deep learning based of artificial neural network along with PLS-SEM to obtain more precise findings. Consequently, proposed models from this study can aid technology makers to formulate strategies accordingly. The current study is grounded on Unified Theory of Acceptance and Use of Technology (UTAUT2) and extends it by adding personal innovativeness and trust in the original model of UTAUT2. Furthermore, this study also investigates the role of personal innovativeness on performance expectancy and price value.

## Literature review

### Constructs mapping

To determine the drivers of behavioural intention (BI), one of the most dominant endogenous variables in the adoption related studies [35], researchers in this study resorted to construct mapping analysis [15]. The exogenous constructs used in the current study are related to several adoption, acceptance and diffusion models and theories, namely the unified theory of acceptance and use of technology (UTAUT) and extended UTAUT (UTAUT2), theory of planned behaviour (TPB), innovation diffusion theory (IDT), and technology acceptance model (TAM). Scores of prior published empirical studies have applied performance outcome constructs such as perceived ease of use and perceived usefulness [24, 36] from TAM. Both performance expectancy and effort expectancy, constructs from UTAUT, have been widely applied by research and considered as the most popular antecedent of behavioural intention [15]. Furthermore, constructs like facilitating conditions and social influence have investigated at rarer instances [15]. Similarly, constructs like price value [37] and hedonic motivation (HM) [38] are investigated but not frequently [14]. Apart from the constructs adopted from the above-mentioned theories and model, there have been several other constructs which have been adopted by previous scholars i.e. perceived risk, personal innovativeness and trust [39, 40].

**Fig 1:**
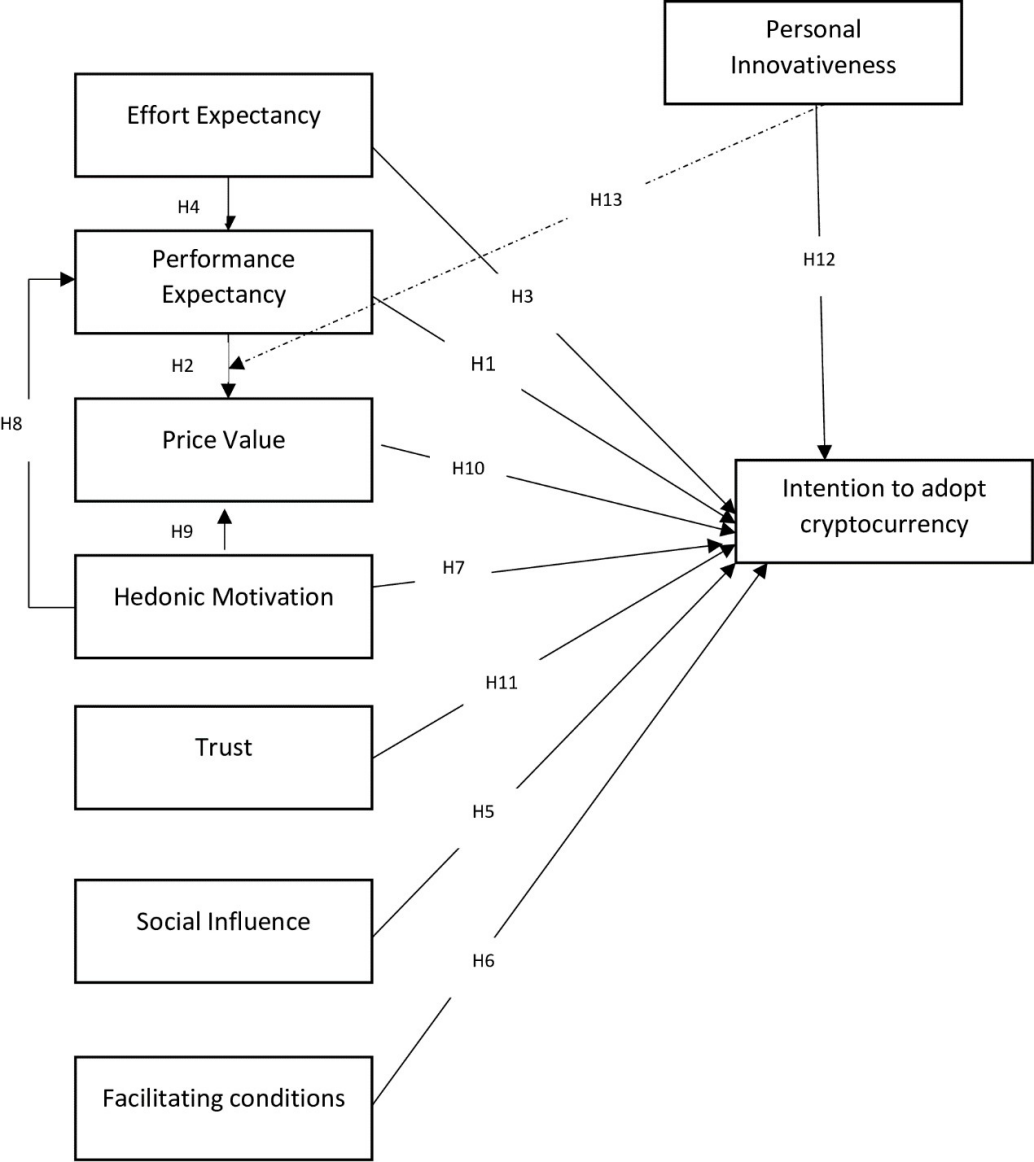
Proposed research framework.

The existing study’s proposed model is grounded on UTAUT2. Also, the researcher extended the UTAUT2 model by including both personal innovativeness and trust in the proposed model [39, 40]. These additional predictors are deemed essential in the context of Malaysia, especially the inclusion of trust in the proposed model primarily in the context of cryptocurrency riddled with the concerns of insecurity, risk and anonymity [5, 6] reflects its sheer need and importance. However, the researchers in this study have not used the construct, i.e. habit, from the original UTAUT2 model. Habit was dropped from the proposed research model on the assumption that as cryptocurrency is in its infancy and still in the process of its developing stage. Moreover, researchers have averred that personal innovations as a construct, should be included in the studies on the basis that people who are more innovative are more inclined to take risks in regards to adopting new and novel technology [41, 42]. Lastly, the researcher also investigated the moderating role of personal innovativeness between users’ intrinsic motivation on perceived usefulness (performance expectancy) and price value, represents a worthwhile research as per the suggestion of Alalwan et al. [43]. To the best of the researchers’ knowledge, no research particularly in Malaysia has investigated the following relationships in the context of cryptocurrency. As shown in Fig 1, performance expectancy, effort expectancy, social influence, facilitating conditions, hedonic motivation, price value, trust and personal innovativeness are used as drivers of consumers behavioural intention to use crypto currency from the perspective of Malaysia. The development of hypotheses is further discussed in detail.

### UTAUT2 –a progression of technology acceptance theory

Extant literature has seen a plethora of theoretical models exploring consumers usage intention and actual adoption of technology. One of the earlier theories in this regard was presented by Davis [44], known as the Technology Acceptance Model and most commonly called TAM. He concluded that users will adopt a technology if they perceive it easy and useful. The Davis [44] model consisted of two main elements: perceived usefulness and perceived ease of use, which have been validated several times by researchers in the domain of information and technology [24, 36]. However, TAM itself alone was found insufficient to determine the adoption of new technology as it ignores the impact of social relations [45].

As the world of information and technology progresses, the Unified Theory of Acceptance and Use of Technology (UTAUT) was introduced by Venkatesh et al. [46], as a new information technology-related theory. UTAUT was constructed after the amalgamation of eight different theories, i.e. theory of reasoned action, TAM, theory of planned behaviour, motivational model, model of PC utilisation (MPCU), combined TAM-theory of planned behaviour, social cognitive theory, and innovation and diffusion theory (IDT). UTAUT consists of four main exogenous variables, i.e. effort expectancy, performance expectancy, facilitating conditions and social influence; and is used to determine behavioural intention. Similarly, both effort expectancy and performance expectancy are closely associated with the primary constructs of TAM, i.e. ease of use and usefulness, respectively.

There is no doubt that UTAUT model has attained higher explanatory power. However, in the eyes of critics, it has been postulated that UTAUT oversees specific factors, which resultantly, is considered as incapable of representing the individuals’ intention to adopt a technology holistically. Moreover, UTAUT was further criticised by academicians, as the UTAUT model was deemed fit for the organisation, due to factors being more related to predict employees’ behavioural intention; and thus making it incapable to gauge consumer-graded innovations such cryptocurrency. It was then that Venkatesh et al. [47] presented a more consumer-centric model which is an extension of the original model and called it UTAUT2. This UTAUT2 included the original UTATA constructs; along with-it authors added three additional variables, i.e. habit, hedonic motivation and price value. In total, seven exogenous constructs to determine the behavioural intention of users. These three additional constructs represent functional, intrinsic and financial benefits [48].

## Hypothesis development and conceptual framework

### Performance Expectancy (PE)

Performance expectancy can be conceptualised as an extent to which a user can receive benefits and values such as customisation, convenience and lack of effort, by the adoption of a specific technology [47]. In the extant literature of information technology (IT)/information systems (IS), performance expectancy has been the key indicator for driving users’ behaviour intention and use behaviour [43, 48]. Notably, it has been established that while using new technology, customers tend to make a cost-benefit rationale valuation [47]. Therefore, especially in the domain of internet banking, it is stated that additional functions and benefits perceived by users using internet banking, adds to the price value of such technology [46].

Similarly, in the eyes of Rogers [49], an innovation to be adopted had to yield any relative advantage or benefits to users. As mentioned earlier, these benefits correspond to performance expectancy. These arguments were further cemented by Ho and Ko [50]; customers perceived usefulness as positively connected to the bank related self-service technology. Therefore, in the context of the study, not only performance expectancy will increase the user’s intention to use cryptocurrency, but it also enhances the price value related to the adoption of cryptocurrency. Thus, based on the above arguments, the following hypotheses are articulated:

**H1**: Performance expectancy has a positive and significant effect on intention to adopt cryptocurrency.
**H2**: Performance expectancy has a positive and significant effect on price value.

### Effort Expectancy (EE)

Effort expectancy, within the context of UTAUT2, is defined as the degree of ease related to consumers’ use of new technology [47] which resultantly, shapes a positive perception and intention towards such innovative medium [37]. Numerous published empirical researches have posited the importance of effort expectancy [43, 48] or its related factor such as perceived ease of use [51, 52]; demonstrate a positive effect on users’ intention to use a technology.

In line with the arguments of Davis [44] which stated that besides the benefits and advantages that an individual gets from the usage of a specific technology, users still find themselves in a mental trade-off process among the efforts needed to apply the technology effectively. Therefore, it was empirically stated that users have higher performance expectancy if they perceive technology easy to use and subsequently, this affects their intention to adopt [43–45]. This argument was further validated when perceived ease of use was found to be a significant predictor of, not only Malaysian] students’, intention to use an online library [53]. Nevertheless, effort expectancy has also proven to be a key indicator in driving Malaysian continuous intentions to use e-government. Furthermore, Herrero and San Martin [53] also empirically stated that effort expectancy has a positive and significant and direct effect on performance expectancy of users. Therefore, in the context of this study, it is expected that Malaysian users are more likely to adopt a cryptocurrency, if it is useful and takes less effort to conduct a task. Consequently, this study articulates the following hypothesis:

**H3**: Effort expectancy has a positive and significant effect on intention to adopt cryptocurrency.
**H4**: Effort expectancy has positive and significant influence on the performance expectancy of using cryptocurrency.

### Social Influence (SI)

Social influence is referred to as a degree of influence that an individual perceives from their loved ones to use technology [47]. Several scholars have extensively investigated the notion of social influence and confirmed its influences on determining individuals’ intention to use and adopt diverse types of technological innovations, e.g. internet banking [43], mobile banking [37], mobile commerce [48], mobile health adoption [54], mobile wallets [15] and wearable technologies [55]. For matters of products and services which are new and in their early stages of development, social influence can play a crucial role, especially where there is lack of information available on the new technology [56]. As the use of cryptocurrency can be considered a solo activity, the positive influence (benefits and advantages) of cryptocurrency from friends or loved ones can influence an individual to adopt such technology. This view was further validated by the Nseke [57] study, which investigated the role of Bitcoins among Africans, and empirically supported the role of social influence in stimulating their intention to use Bitcoin. Therefore, based on the arguments, the researcher articulated the following hypothesis:

**H5**: Social influence has a positive and significant effect on intention to adopt cryptocurrency.

### Facilitating Conditions (FC)

Facilitating conditions refer to the perception of an individual who believes that for using a novel technology, the technical infrastructure is already there to facilitate users [47]. Extant literature is replete with studies which have empirically established that facilitating conditions is the fundamental driver of users’ intention to adopt a technology [45, 46, 99]. Thus, in the context of our research, facilitating conditions echo the outcome of necessary resources available (interconnectivity and flexibility of using it on any IT device such as smartphone, tablets and others) and the essential knowledge needed to perform a transaction through cryptocurrency. Therefore, based on the above arguments, the current study articulates the following hypothesis:

**H6**: Facilitating conditions have a positive and significant effect on intention to adopt cryptocurrency.

### Hedonic Motivation (HM)

According to Venkatesh et al. [47], hedonic motivation refers to the feelings of joy, amusement or happiness driven by the usage of a specific technology. These emotions which capture the hedonic motivation of users, resultantly becomes the substantial cause of individuals accepting a technology [58, 99]. Besides, captivatingly, cryptocurrency usage could be conceived as part of contemporary life; resultantly enhancing additional value to the fraction of users who actively seek innovation and novelty [59]. Furthermore, research has also established that users who use a technology which provides fun, enjoyment and amusement, are more likely to use it more effectively. Thus, adding extra value to such technology (e.g. price value, performance expectancy). During their research, Alalwan et al. [43] established the empirical results that if user’s hedonic motivation using internet banking is high, users’ will perceive higher benefits which will impact their performance expectancy, and simultaneously augment the price value pertaining to internet banking, as such, leading to the making of the following hypothesis:

**H7**: Hedonic motivation has a positive and significant effect on intention to adopt cryptocurrency.
**H8**: Hedonic motivation has a positive and significant effect on performance expectancy of using cryptocurrency.
**H9**: Hedonic motivation has a positive and significant effect on price value of using cryptocurrency.

### Price Value (PV)

Price value is conceptualised as the cognitive balance between the fiscal cost and the benefit of using a technology [47]. Unlike in an organisational setting, the cost of any new technology usage is borne by the user. Therefore, the user will contemplate the cost-benefit evaluation before deciding to adopt a technology. The user’s inclination to adopt a technology certainly increases when the price value surpasses the financial cost [45]. Previous literature has successfully demonstrated empirically, the significance of price value on user adoption intention [37, 43]. In the extant literature of marketing, to investigate the perceived value of any products or services, the balance between the cost/price evaluation is mostly made with the quality of the product or services acquired, or the benefits perceived by users [37, 43, 99]. Therefore, based on the above arguments, it hypothesised that:

**H10**: Price value has a positive and significant effect on intention to use cryptocurrency.

### Trust (TR)

According to Koksal and Penez [60], trust is a subjective disposition; and it is referred as an extent to which a user believes that the service provider (trustee) is safe, and it is secure to make any transaction with them. Prior research has found trust to be a critical indicator of behavioural intention to adopt a technology [37, 61, 62]; especially in the mobile banking literature, where two types of trust have been eminent, namely trust in the technology or the medium and institutional trust [60]. The later exhibits the trust among the financial service providers and users, based on their previous experience. In the context of cryptocurrency, this is not applicable, just as in any novel financial technology. Therefore, based on the above argument, the following hypothesis is proposed:

**H11**: Trust has a positive and significant effect on intention to adopt cryptocurrency.

### Personal Innovativeness (PI)

As per Rogers [49] innovation diffusion theory, both individual differences and personality characteristics influence people’s adoption of novel, unique and original ideas, as well as objects or practices. Based on this idea, researchers [42, 63] conceptualised personal innovativeness as the degree to which an individual believes that he/she is positively inclined towards the use of innovative and novel technologies. According to Agarwal and Prasad [42], personal innovativeness exhibits the individual’s intrinsic innovative personality-related characteristic, which in view of Shaw and Sergueeva [48], is a risk-taking trait in an individual. Similarly, individuals who possess higher risk-tolerance are more likely to attempt to use a new technology since they have more positive beliefs about technology usage [64]. Overall, individuals with more innovative personalities are apt to have a higher intention to use new technology [48]. Several empirical studies have established a significant relationship between personal innovativeness and behavioural intention [65, 66]. In the context of this study, we posit that the impact of performance expectancy on perceived value will be moderated positively by personal innovativeness. This means that users who are extra innovative will try and adopt cryptocurrency usage as new technology in their daily lives, even when they do not perceive the value to be high (e.g. price value). As proven by earlier literature that early adopters tend to accept fewer features and embrace more risks. Therefore, based on the following arguments, this study articulates the following hypotheses:

**H12**: Personal innovativeness has a positive effect on intention to adopt cryptocurrency.
**H13**: Personal innovative will positively moderate the relationship between performance expectancy and price value.

### Research model

This study proposed research model is shown in Fig 1.

## Research methodology

### Ethical approval

With regard to ethical approval, this study did not seek ethical approval because this study was observational in nature and no formal approval of the Institutional Review Board of the Local Ethics Committee was necessary in the absence of the involvement of therapeutic drugs. Despite these challenges, the study was informed all subjects and participation was entirely voluntary. The cover letter of the survey indicated that the confidentiality of all participants will be preserved along with the details that authors will take this as a consent if the respondents want to participate in this survey. No juveniles were included in this research. It was undertaken in the context of the Helsinki declaration.

### Data collection

To achieve the aim and objectives of the research, the researchers resorted to online surveys for data collection. According to Bhattacherjee [67], the use of online survey encompasses several benefits, i.e. economical, time saving and wider audiences. Also owing to the lack of sample frame, this study adopted a non-probability purposive sampling method to collect data. According to Sekaran and Bougie [68], this method is regarded as the most effective method in order to get views of potential respondents regarding the studied phenomenon. Data collection took place in 2019, in Malaysia. The link of the online questionnaire was developed by using google survey forms which were disseminated through the use of social media, i.e. Facebook. An online survey of 38 questions, excluding demography, had been categorised into three sections, see Table 1. Section A contained a cover letter explaining the motive of the study. Section B included respondents’ demographic information, while Section C of the online survey included measurement items of each construct used in the study, see Table 2. Social media was chosen as the medium for data collection owing to its massive usage among Malaysian citizens. According to Statista [69], the total of Malaysian Facebook users is expected to grow to 27.34 million in 2025, compared to previous years of 23.0 million in 2019, and 24.85 million in 2020. From the total 350 collected responses, 36 were discarded due to incompleteness, leaving researchers with a final 314 responses for further analyses. In regard to the sample size, Kline [70] postulated that an inadequate sample size might adversely affect the generalisability of study findings. For the estimation of minimum sample size, several rules of thumb have been prescribed. Deb and David [71], suggested an item to respondent to the rule of thumb. In their view, 1:4 and 1:10 (item: response) must be adequate for any research. Given that, a sample size of 152:380 for a study with 38 total items, must be satisfactory. Hence, in view of the above discussion, this study’s sample size is acceptable. All the constructs except for the dependent variable of the study, i.e. behavioural intention was measured on a five-point Likert scale ranging from 5 i.e. strongly agree to 1 i.e. strongly disagree. Whereas, the seven-point Likert scale was used for dependent variables ranging from 1 i.e. strongly disagree to 7 i.e. strongly agree. The studied questionnaire was further validated by academic experts from technology adoption domains as a pre-test. Upon the request of the academic experts, the questionnaire was pilot tested on a small sample. Pilot test findings were satisfactory as reliability of all the constructs were found to be more than 0.70.

**Table 1:** Profile of respondents.

Variable	Frequency	(%)
***Nationality***		
Malaysian	314	100%
***Age***		
18–25	28	8.9%
26–35	180	57.3%
36–45	75	23.9%
46 and above	31	9.9%
***Living State***		
Penang	98	31.2%
Selangor	123	39.2%
Johor	93	29.6%
***Gender***		
Female	139	44.3%
Male	175	55.7%
***Marital Status***		
Single	149	47.5%
Married	165	52.5%
Other	0	0%
***Gross Monthly Income***		
Below RM 2,000	24	7.6%
RM 2,000—RM 5,000	130	41.4%
RM 5,001—RM 10,000	113	36%
RM 10,001 and above	47	15%
***Highest Education Level***		
High School	31	9.9%
Diploma	51	16.2%
Bachelor’s degree	201	64%
Master’s degree	30	9.6%
PhD	1	0.3%

**Table 2:** ANOVA: Deviation from linearity.

		Sum of Squares	df	Mean Square	F	Sig	Linear
BI*PE	Deviation from Linearity	46.707	15	3.114	2.217	.006	**NO**
BI*EE	Deviation from Linearity	14.234	11	1.294	1.087	.371	YES
BI*FC	Deviation from Linearity	8.042	15	.536	.627	.852	YES
BI*HM	Deviation from Linearity	22.153	11	2.014	1.574	.105	YES
BI*PV	Deviation from Linearity	18.511	11	1.683	2.045	.024	**NO**
BI*TR	Deviation from Linearity	6.847	15	.456	.599	.875	YES
BI*PI	Deviation from Linearity	28.655	10	2.865	1.375	.191	YES
BI*SI	Deviation from Linearity	14.234	11	1.294	1.087	.371	YES

Note: BI: Behavioural intention; EE: Effort expectancy; pe: performance expectancy; FC: Facilitating condition; HM: Hedonic motivation; pv: price value; TR: Trust; PI: Personal innovativeness; SI: Social influence.

### Data analysis

Contrary to previous empirical studies which had employed shallow ANN approach along with structural equational modelling [29–31], the current study has adopted the deep leaning dual-stage SEM-ANN approach to confirm the legitimacy of hypothesised relations in the research model [33]. This methodology consists of two phases. In the initial phase, PLS-SEM was adopted to recognise the substantial effects of predictors. In the subsequent phase, researchers implemented deep ANN architecture to identify the rank of constructs via sensitivity analysis.

#### PLS-SEM

The proposed research model is tested using the PLS-SEM method. PLS-SEM is regarded as useful in comparison to covariance-based SEM (CB-SEM), due to several reasons, such as its ability to test complex models [72, 73], handle small samples and non-normal data (Hair et al. [76], for further details), and is predominantly very useful for testing the moderating effect between constructs [73, 74]. Furthermore, after reviewing the work of Ronko and Evermann [75], it was suggested that PLS-SEM is more efficient than CB-SEM, particularly in the quest of finding the true model [76]. Analysis via PLS-SEM consists of two components: assessment of measurement model and structural model [77–79]. Outer model measures the reliability and validity, whereas the inner model tests the strength of the associations among variables [76, 79].

#### Artificial Neural Network (ANN)

Once both the components of PLS-SEM are validated, artificial neural network (ANN) is employed to assess, complement and authenticate the PLS-SEM analyses. According to the Henseler et al. [80] research, which is predictive in nature, the use of ANN will be beneficial. ANN is referred to as a processor which is capable of accumulating knowledge and to putting it to use. It is known as a computational procedure that can imitate the method of transferring information in the human brain. Furthermore, it is regarded as a function approximation tool which is deemed fitting in circumstances where the nature of the interaction between output(s) and input(s) is nonlinear and complex. According to Simpson [81], ANN consists of three main mechanisms: network architecture, transfer function and learning rule. These three components are categorised in the following subsections, i.e. recurrent network, radian basis and feed-forward network [82]. Feedforward multi-layer perceptron (MLP) neural network, which consists of several layers, such as input and output, is most widely applied. Both these input and output layers are connected via hidden nodes. Weights represent each hidden node. Fundamentally, the input layer contains several neurons (independent variables) which obtain raw data and passes it to the hidden layers in the shape of synaptic weights. The output of each hidden layer is reliant on the choice of activation function, among which the most frequently used is the sigmoidal function [28, 29]. Furthermore, multi-layer neural network models are observed as very complicated and very powerful, with the capability to resolve intricacies in higher-order models. Thus, the researcher in this study adopts multi-layer perceptron neural networks to test and train the proposed research model.

## Results

### Descriptive results

A total of 350 responses were collected. Out of 350, only 314 were found and deemed fit for the current study’s descriptive analysis using SPSS. From a sample of 314 respondents, approximately 55.7% were males, and the vast majority had a Bachelor level of education (64%) and were aged between 26–35 years old (57.3%). In addition, out of the three states, majority of the participants were from Selangor (39.2%); similarly, most of the respondents also happened to be married (52.5%) see Table 2.

### Common Method Bias (CMB)

To also cater the common method bias (CMB), this study followed the recommendations of Padsakoff et al. [83] by rigorously applying both statistic and procedural remedies. In regard to procedural measures, the researcher strategically employed the application of multi-scale to cater to the common method variance, as suggested by Podsakoff et al. [83]. Concerning statistical remedies, this study applied several remedies for rigorous testing. Firstly, Harman single factor test was used. Findings reveal that the total variance examined by a single factor was found to be 44% which was less than 50% [83]. Moreover, even though Harman’s single factor analysis is most commonly applied, researchers have demanded that it is not appropriate to engage with common method variance [84]. Therefore, following Bagozzi et al. [85], researchers used correlation matrix to further validate it. As per correlation matrix results, no value was found with more than 0.90 among the constructs; thereby confirming that CMB was not regarded as the major concern.

### Linearity test

Researchers also investigated the linearity between the studied constructs by applying the p-value of the deviation from linearity with ANOVA test in SPSS. The findings from Table 2 clearly illustrates that the studied model has a mix of both non-linear and linear associations among the target construct and its predictors. For further validation, researchers decided to conduct Ordinary Least Squares test (OLS) and the findings in Table 3, with the help of p-values affirms that there are indeed linear relationships.

**Table 3:** Ordinary Least Squares (OLS) test.

Model	Sum of Squares	Df	Mean Square	F	Sig
Regression	297.719	1	297.719	200.229	.000^b^
Residual	463.909	312	1.487		
a. Dependent Variable: BI					
b. Predictors: (Constant), PE					
Regression	495.455	1	495.455	580.759	.000^b^
Residual	266.172	312	.853		
a. Dependent Variable: BI					
b. Predictors: (Constant), PV					

### Assessment of outer model

As the first component of PLS analysis, validation of measurement can be made by assessing construct reliability (composite reliability-CR), convergent validity (factor loadings and average variance extracted-AVE) and discriminant validity (hetrotrait-monotrait ratio-HTMT) [86]. Findings in Table 4 reveal that factor loadings, AVE and CR are more the suggested values i.e. 0.707, 0.5 and 0.7, respectively [86].

**Table 4:** Summary of constructs with measurement items.

Construct	Origin	Definition	Loadings	CR	AVE
BI	Hossain et al. [100]	• I intend to use Cryptocurrencies in the future.	0.893	0.926	0.807
		• I will always try to use Cryptocurrencies.	0.891		
		• I plan to continue use Cryptocurrencies frequently.	0.911		
PI	Hossain et al. [100]	• If I hear about a new information technology, I would look for ways to experiment with it.	0.914	0.950	0.864
		• Among my peers, I am usually the first to try out new information technologies.	0.943		
		• In general, I am not hesitant to try out new information technologies.	0.931		
TR	Slade et al. [40]	• I trust Cryptocurrencies to be reliable.	0.947	0.978	0.918
		• I trust Cryptocurrencies to be secure.	0.951		
		• I believe Cryptocurrencies are trustworthy.	0.967		
		• I trust Cryptocurrencies.	0.967		
PV	Beza et al. [99]	• Cryptocurrencies are reasonably priced.	0.89	0.913	0.779
		• Cryptocurrencies is good value for the money.	0.854		
		• At the current price, Cryptocurrencies provides a good value.	0.902		
HM	Beza et al. [99]	• Using Cryptocurrencies is fun.	0.954	0.971	0.919
		• Using Cryptocurrencies is enjoyable.	0.965		
		• Using Cryptocurrencies is very entertaining.	0.956		
FC	Beza et al. [99]	• I have the resources necessary to use Cryptocurrencies.	0.867	0.897	0.685
		• I have the knowledge necessary to use Cryptocurrencies.	0.843		
		• Cryptocurrencies is compatible with other technologies I use.	0.814		
		• I can get help from others when I have difficulties using Cryptocurrencies.	0.785		
SI	Alalwan et al. [43]	• People who are important to me think that I should use Cryptocurrencies.	0.876	0.939	0.838
		• People who influence my behavioral think that I should use Cryptocurrencies.	0.933		
		• People whose opinions that I value prefer that I use Cryptocurrencies.	0.936		
EE	Alalwan et al. [43]	• Learning how to use Cryptocurrencies is easy for me.	0.933	0.967	0.879
		• My interaction with Cryptocurrencies is clear and understandable.	0.93		
		• I find Cryptocurrencies easy to use.	0.961		
		• It is easy for me to become skillful at using Cryptocurrencies.	0.926		
PE	Alalwan et al. [43]	• I find Cryptocurrencies useful in my daily life.	0.93	0.961	0.861
		• Using Cryptocurrencies increases my chances of achieving tasks that are important to me.	0.943		
		• Using Cryptocurrencies help me accomplish tasks more quickly.	0.93		
		• Using Cryptocurrencies increase my productivity.	0.908		

Note: CR: composite reliability; AVE: average variance extracted. BI: behavioural intention; EE: effort expectancy; PE: performance expectancy; FC: facilitating conditions; HM: hedonic motivation; PI: personnel innovativeness; PV: price value; SI: social influence; TR: trust.

Along with convergent validity and construct reliability, this study also examined discriminant validity by using HTMT ratios by benchmarking Kline [87] recommendation. Findings in Table 5 disclose that all the construct HTM values were lower than 0.85 [72], therefore, no serious concerns were established pertaining to discriminant validity. As such it can be concluded that the measurement model was validated.

**Table 5:** HTMT values.

	BI	EE	FC	HM	PE	PI	PV	SI	TR
**BI**									
**EE**	0.79								
**FC**	0.09	0.04							
**HM**	0.68	0.69	0.04						
**PE**	0.62	0.54	0.04	0.61					
**PI**	0.83	0.79	0.08	0.73	0.62				
**PV**	0.59	0.50	0.05	0.50	0.48	0.47			
**SI**	0.72	0.78	0.04	0.68	0.65	0.77	0.44		
**TR_**	0.83	0.82	0.03	0.71	0.56	0.85	0.51	0.75	

Note: BI: behavioural intention; EE: effort expectancy; PE: performance expectancy; FC: facilitating conditions; HM: hedonic motivation; PI: personnel innovativeness; PV: price value; SI: social influence; TR: trust.

### Assessment of multi-collinearity

This study also investigated the issue of lateral collinearity. According to Kock and Lynn [88], although the study has met discriminant validity during the assessment of outer model in preceding section, lateral collinearity issues can sometimes mislead the researcher; therefore, it was decided to investigate. Variance inflated factor (VIF) value of 5 or higher shows possible collinearity matter as suggested by Hair et al. [89]. However, findings in Table 6 shows that no concerns pertaining to the multi-collinearity was detected as the VIF values were lower than 5.

**Table 6:** Path coefficients and hypotheses testing.

Hypothesis	Relationship	Std Beta	T-Value	P-Values	Decision	f^2^	VIF
H1	PE -> BI	0.090	2.083	0.019**	Supported	0.014	1.855
H2	PE -> PV	0.213	3.938	0***	Supported	0.038	1.660
H3	EE -> BI	0.170	2.078	0.019**	Supported	0.027	3.333
H4	EE -> PE	0.237	3.434	0***	Supported	0.050	1.754
H5	SI -> BI	0.034	0.59	0.278	Not Supported	0.001	2.808
H6	FC -> BI	-0.053	1.389	0.083	Not Supported	0.009	1.022
H7	HM -> BI	0.008	0.134	0.447	Not Supported	0.494	2.372
H8	HM -> PE	0.422	5.955	0***	Supported	0.000	1.754
H9	HM -> PV	0.261	3.367	0***	Supported	0.045	2.091
H10	PV -> BI	0.146	3.408	0***	Supported	0.047	1.417
H11	TRUST_ -> BI	0.299	3.958	0***	Supported	0.071	3.939
H12	PI -> BI	0.230	2.671	0.004***	Supported	0.098	3.562
Moderating Effect of Personal Innovativeness							
H13	Moderating Effect	0.136	2.051	0.02**	Supported	0.027^a^	

Note: EE, effort expectancy; FC, facilitating factor; HM, hedonic motivation; PI personal innovativeness; PE, performance expectancy; PV, price value; SI, social influence; BI, behavioural intention

*p < .5

**p < .01

***p < .001.

^an^ effect size calculated manually.

### Assessment of inner model

**Fig 2:**
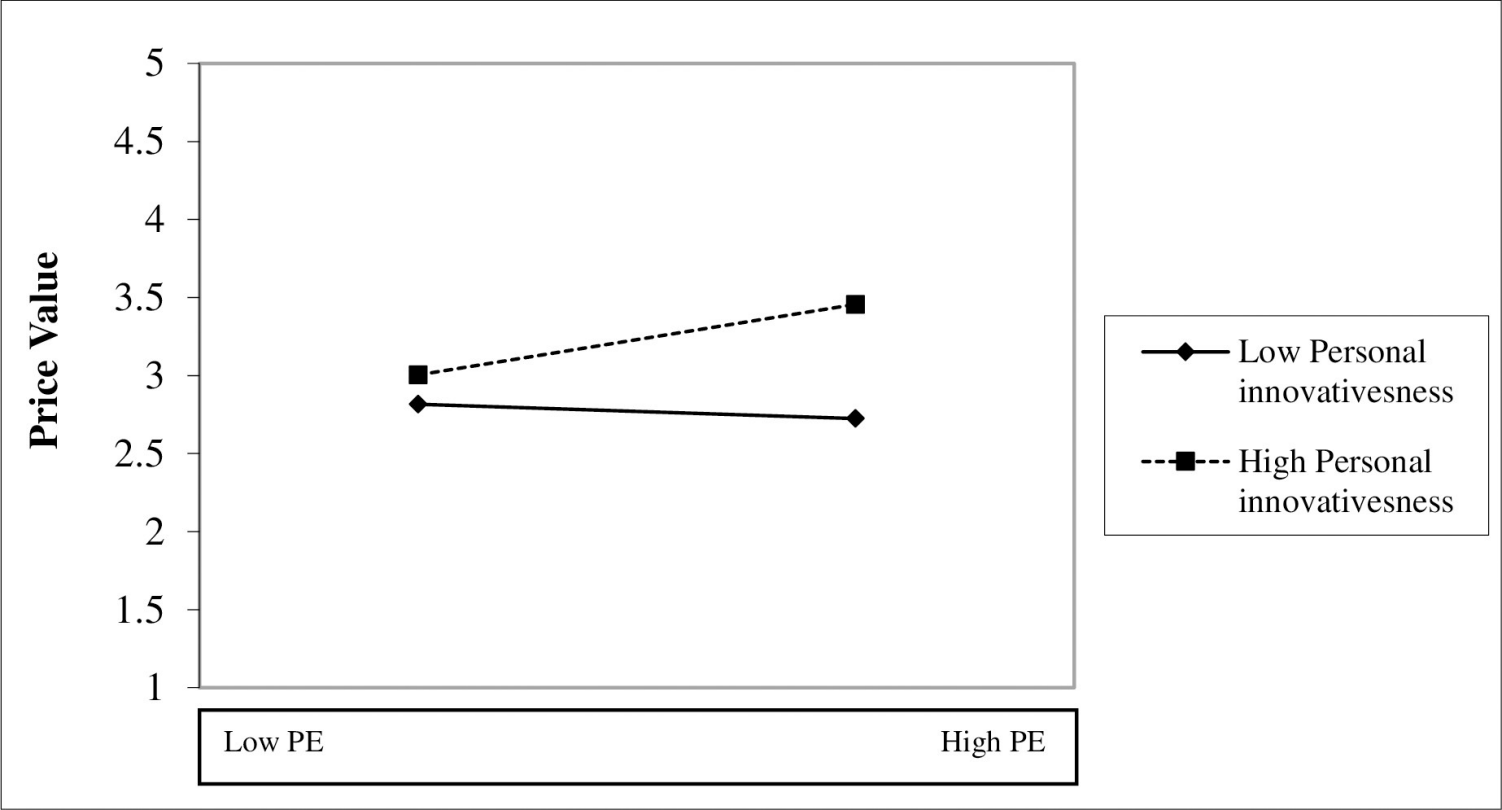
Interaction effect of personal innovativeness on performance expectancy and price value.

Once the measurement model is analysed, to examine the proposed relationships in the studied model, structural model analyses were conducted. For the validation of the proposed hypotheses, bootstrapping procedure with 5000 iterations were applied, following suggestions of Hair et al. [86]. Table 6 reveals the results of hypotheses. Moreover, current study factors yielded 28.0%, 36.5% and 68.20%, of the variance towards price value, performance expectancy and behaviour intention, respectively. Following the Cohen [90] guidelines effect size (f^2^) were also calculated, see Table 6. Besides, co-efficient of determination (R^2^) and effect size (f^2^) this study also calculated predictive relevance (Q^2^). Findings of this study revealed that the Q^2^ value for all the endogenous construct were more than zero (BI: 0.509), PE: 0.293) and PV: 0.196) [91]. Furthermore, Fig 2 illustrates that personal innovativeness positively moderates the relationship between performance expectancy and price value. Besides, as recommended by Shmueli et al. [92] proposed PLSPredict, this study applies the PLS Predict process to validate further predictive relevance of the study. Findings from Table 7 reveal that our model has strong predictive power.

**Table 7:** PLS predict.

Items	LM	PLS	LM-PLS	Q^2^ Predict
	RMSE	RMSE		
**BI3**	0.114	1.12	0.114	0.528
**BI1**	0.114	0.998	0.114	0.532
**BI2**	0.115	1.115	0.115	0.464
**BI**	--	0.612	--	0.631

Note: LM: Linear model; RMSE: Root Mean square errors; PLS-SEM: Partial least squares-structural equation modelling.

### Moderating effect of personal innovativeness

To investigate the moderating effect of personal innovativeness on performance expectancy and price value, the researcher applied orthogonalizing approach for interaction term which should be preferred than product indicator and two-stage approach [28]. The result demonstrated that personal innovativeness positively moderates the relationship (β = 0.115; p < .05), thus hypothesis H13 was supported. Using the formula mentioned below, the effect size of the moderating relationship was estimated.

F^2^ = (R^2^ with moderator–R^2^ without moderator) / (1- R^2^ with moderator).

R^2^ values for price value, including the moderator, was 0.28, and the excluding moderator was 0.26. Thus, these according to the formula, the effect-size values were calculated, i.e. 0.03, which is considered a small effect [90].

### Artificial neural network analysis

**Fig 3:**
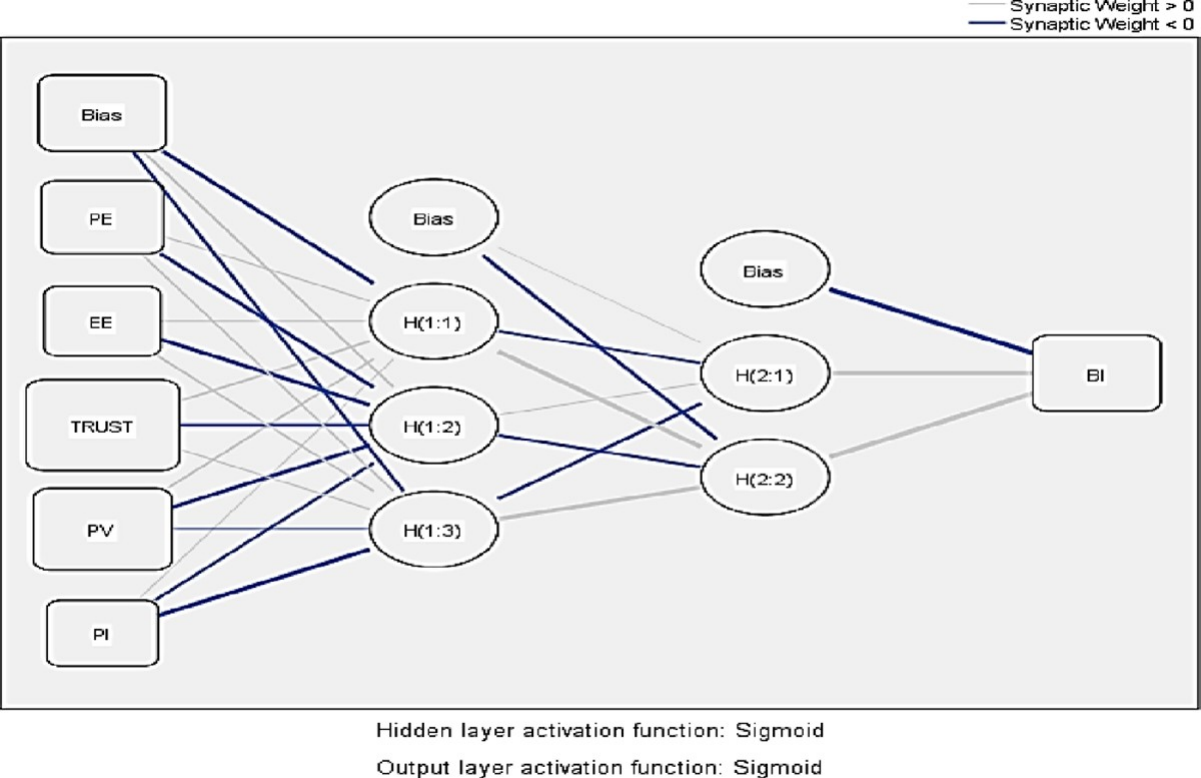
Deep ANN model for the intention to adopt cryptocurrency. EE, effort expectancy; PI, personal innovativeness; PE, performance expectancy; PV, price value; TR, trust.

ANN analysis was performed through the most extensively used statistical software, i.e. SPSS 23. ANN analysis considers the significant predictors from PLS-SEM results. Owing to several endogenous constructs, this study only focuses on the dependent variable of the study, i.e. behavioural intention (with highest numbers of predictors); hence, there will be only one deep ANN model (see Fig 3 as an example). The ANN model is made up on one output neuron, i.e. dependent variable and several input neurons (significant indicators to behavioural intention), such as effort expectancy, performance expectancy, price value, trust and personal innovativeness. Two-hidden layer deep ANN architecture was employed with an aim to allow deeper learning to take place for each of the output neuron node [33] instead of single hidden layer model which are referred as shallow ANN approach. This study uses sigmoid function as the activation function for both output and hidden neurons. Also, the range for both input and output neurons are normalised between [0,1] to enhance the performance of the current model [93]. Furthermore, to avoid the overfitting in ANN models, a ten-fold cross-validation procedure was applied with a ratio of 10:90 for both testing and training data, respectively. Pertaining to the accuracy of neural network models, root mean square of errors (RMSE) is recommended. The RMSE values of ANN model of this study for both training and testing data (see Table 8) ranges from 0.084 to 0.103 and 0.071 to 0.103, respectively. Owing to the very minute differences between RMSE values and the standard deviation for both training and testing data, researchers can predict that the proposed researcher model achieves higher accuracy with ANN.

**Table 8:** RMSE values.

	RMSE (Training) (BI)	RMSE (Testing) (BI)	R^2^ PLS-SEM	R^2^ ANN
	0.087	0.065	68.20%	74.00%
	0.108	0.076		
	0.093	0.071		
	0.088	0.070		
	0.081	0.072		
	0.093	0.096		
	0.090	0.075		
	0.085	0.076		
	0.086	0.077		
	0.083	0.065		
**SD**	**0.007**	**0.008**		
**Mean**	**0.082**	**0.074**		

Note: BI, behavioural intention. SD, standard deviation.

Furthermore, to calculate the relative normalized importance, the mean of each predictor is used against the highest mean value, expressed in percentage. Table 9 provides the details of the normalized importance and the mean importance of all the predictors used in ANN modelling. According to Table 9, the result of the sensitivity analysis displays that trust is the most vital predictor of behavioural intention to use cryptocurrency, followed by price value and effort expectancy. Moreover, personal innovativeness was found to be the least predictor to develop the behaviour intention among Malaysian for the adoption of cryptocurrency which contradicts results of PLS-SEM significant constructs based on f^2^ values mentioned in Table 9. Furthermore, in the quest to validate the performance of ANN models, it was suggested by Leong et al. [94] to calculate the goodness of fit which is comparable to R^2^ from PLS findings. Findings in Table 8 clearly depicts that our proposed research model of the study has shown higher predictive power as compared to R^2^ results based on PLS-SEM; thereby, meeting one of the objectives of the study. The difference of result can be validated by the better predictive accuracy while using deep ANN architecture and also the non-compensatory nature of the deep learning dual stage hybrid PLS-SEM & ANN method. These results further state that studied endogenous variables are better explained by ANN analysis in comparison to SEM analysis. At the same time, researchers opine that the current study outcome is due to the superiority of two-hidden deep learning architecture and its capacity to capture non-linear associations among the variables.

**Table 9:** Sensitivity analysis.

Sensitivity Analysis (Importance)	PE	EE	TR	PV	PI
**ANN1**	0.227	0.150	0.234	0.283	0.105
**ANN2**	0.118	0.237	0.281	0.251	0.113
**ANN3**	0.137	0.180	0.303	0.284	0.096
**ANN4**	0.163	0.133	0.313	0.290	0.101
**ANN5**	0.151	0.169	0.317	0.281	0.082
**ANN6**	0.115	0.190	0.436	0.200	0.060
**ANN7**	0.153	0.170	0.314	0.280	0.084
**ANN8**	0.166	0.189	0.291	0.226	0.128
**ANN9**	0.160	0.166	0.322	0.284	0.078
**ANN10**	0.147	0.169	0.33	0.226	0.124
**Mean Relative Importance**	**0.154**	**0.175**	**0.315**	**0.261**	**0.097**
**SD**	**0.030**	**0.026**	**0.048**	**0.031**	**0.020**
**Normalised Relative Importance%**	**49%**	**56%**	**100%**	**83%**	**31%**
**Ranking**	**4**	**3**	**1**	**2**	**5**

Note: SD, standard deviation. EE, effort expectancy; PI personal innovativeness; PE, performance expectancy; PV, price value; TR, trust.

## Discussion

This research sought to inspect the crucial factors that may influence the adoption of cryptocurrency in Malaysia through the modification of the UTAUT2 with trust and personal innovativeness. The researcher also investigated the role of personal innovativeness between price value and performance expectancy. Researchers excluded the construct habit from the original UTAUT2 model on the assumption that habit is regarded as the notion of previous actions and it is regarded as the degree to which an individual is inclined to do a particular behaviour routinely or repeatedly [47]. In addition, Beh at al. [45] narrated that practice is supposed to be a precondition of habit formation. As cryptocurrency is in the infancy stage; therefore, habit construct was dropped from the proposed conceptual model of this study. Theoretically, the obtained empirical findings contrasted from the original UTAUT2 model. AS per the findings of this study, all relationship except facilitating condition, hedonic motivation and social influence (H5, H6 and H7) were found statistically significant. Also, the personal innovativeness as the moderator was found to have a positive and influence moderating effect between the price value and performance expectancy.

According to the findings of the study, user’s behavioural intention to use cryptocurrency was positively influenced by performance expectancy (H1: β = 0.121) and price value (H2: β = 0.213). This indicates that Malaysian users are expected to have a stronger intention to adopt cryptocurrency if they believe that the use of this technology is useful and enables them to complete a task successfully. Moreover, the results of this study have also strongly supported the relationship between performance expectancy and price value pertaining to the usage of cryptocurrency. That is to say that users observe adopting cryptocurrency as a beneficial technology making a significant impact in their everyday lives with numerous benefits (e.g. convenience, timesaving and efficiency); and also believe that adopting cryptocurrency is more valuable than the cost paid for this technology. Findings of this research are in line with earlier related literature, e.g. Alalwan et al. [43] and Beh et al. [45] and Gupta et al. [62]. As expected, effort expectancy has shown positive and significant influence on users’ behavioural intention (H3: β = 0.170); however, an insignificant effect of it was established on users’ effort expectancy (H4: β = 0. 237). The results of this study are in line with Beh et al. [45] and Herrero and San Martin [53]. This highlights that to entice user intention to adopt cryptocurrency, it is insufficient to advance the performance of the cryptocurrency; instead, it is also vital to make the use of cryptocurrency easy and hassle-free.

Strangely and contrary to the findings of past studies [56, 57, 65], insignificant relationships were established between social influence and behavioural intention to adopt cryptocurrency among Malaysian users’ (H5: β = 0.034). The findings of our study were further reflected in other papers where there was no effect of social influence on user’s conclusion to embrace a particular technology (cryptocurrency), as demonstrated in diverse circumstances such as mobile banking and internet banking [37, 43]. This highlights that opinions from closed and loved ones, such as family and peers, have no influence on their intention to use cryptocurrency. One of the possible explanations for the findings may be that the use of cryptocurrency for financial matters and the information relating to it, is regarded as a solo act and very private to users. Therefore, this limited sharing of information with friends or family, lessens the impact of a social network. Another explanation behind the results may be that some respondents in our study are early adopters who are considered reluctant to be influenced by the opinions of others. According to Läpple and Van Rensburg [95], early adopters consumers tend to be young but with higher education, a similar case was observed in this research with respondent profiles (18–35 years old: 66.2%; Bachelor’s degree and above: 73.9%).

The results of the study also reveal that facilitating condition has an insignificant effect on behavioural intention (H6: β = -0.053). The findings of this research are in line with Shaw and Sergueeva [48], Merhi et al [37] and Herrero and San Martin [53]. The explanation behind the results of this study may be that facilitating conditions pertaining to the use of cryptocurrency is easily obtainable; a smartphone, tablet or laptop connected to the internet are the tools required to complete a task. According to Malaysian communications and multimedia commission [96], 87.4% of the total population are internet users; similarly, 9 out of 10 people use the smartphone. Moreover, according to Statista [97], 84.8% of Malaysians aged 18–34 are smartphone users. Therefore, access of technology to the majority of our study sample is pretty modest, as they seem to have vast experience with these devices. The researcher posits that these reasons may have led us to find the insignificant association between the studied constructs.

Moreover, the results of our study also found insignificant association among behavioural intention and hedonic motivation (H7: β = 0.008) whereas the direct relationship of hedonic motivation to performance expectancy (H8: β = 0.422) and price value (H9: β = 0.261) were found to have significantly positive effect. In relation to the direct relation to behavioural intention, the result of our study was contrary to the findings of Shaw and Sergueeva [48] and Beh et al. [45], nonetheless in line with the findings of Merhi et al. [37], Owusu Kwateng et al [61] and Gupta et al [62]. The possible reason behind this insignificant finding may be due to the users’ perception of cryptocurrency usage as a beneficial service, as opposed to being a channel of amusement; a suggestion reinforced by the observed performance expectancy which in this study, is a significant factor in the Malaysian adult intention to adopt cryptocurrency. Furthermore, this research also provided statistical shreds of evidence approving the significance of hedonic motivation on both performance expectation and price value. These results indicate that users who perceive using cryptocurrency inclusive of entertainment and enjoyment, perceive it as highly useful and as having a price value.

Furthermore, the results of the study also exhibit that the direct relationship of price value (H10: β = 0.146), trust (H11: β = 0.299) and personal innovativeness (H12: β = 0.230) to adopt cryptocurrency was found to be positively significant. The findings of our study are in line with Merhi et al. [37], Alawan et al. [43], Farooq et al. [65] and Dutta et al [66]. Trust, in this study, has proved to be the most potent effect in finding the intention to adopt cryptocurrency. This indicates that there is a need for adequate safety measures to take steps in future which can eradicate any risk and threat involved in it, which could inhibit potential future users. In the extant literature of novel technology, trust has emerged as the most influential factor in several studies, especially pertaining to mobile/internet banking [37]. Riffai et al [59] further validated the argument by stating that due to the intricate nature of electronic financial transactions, trust plays a vital role.

According to the results, personal innovativeness as a moderator has worked well in this study. The path analysis for performance expectancy to price value was moderated significantly (H13: β = 0.136); however, the effect was weak as per Cohen’s [90] threshold. Moderation result indicates that innovative users will try use and adopt the use of cryptocurrency even when do not see the value to be high (e.g. price value).

### Contribution to the theory

The findings of the existing research have overwhelmingly advanced the extant literature on the information system, internet banking and especially to the cryptocurrency-related literature by enhancing the current body of knowledge concerning such imperative situation of attention, as well as providing valuable understanding for both academicians and practitioners. UTAUT2 was chosen as the appropriate model because it is explicitly suggested to elucidate the process of adoption novel technologies [47]. Therefore, substantial input was taken by the existing study as one of the very few studies which have enhanced the validity to investigate and expound the determinants that can impact the intention to adopt cryptocurrency on Malaysian customers as a developing country. Notwithstanding that UTAUT2 model has already a satisfactory view towards adopting new technology, proposing a model which is efficient enough to interpret users’ intention towards a sensitive technology like cryptocurrency calls for integrating new factors and the modification of the relationship of the original UTAUT2 model [47].

Therefore, the current study extends the original UTAUT2 model by including constructs like trust and personal innovativeness; at the same time, the researcher has excluded the construct habit from the proposed model. Consequently, the study results demonstrated that both trust and personal innovativeness possess a noteworthy effect on users’ intention to adopt cryptocurrency, especially from the perspective of a developing nation like Malaysia. Pertaining to modification of UTAUT2 original model, the current study has changed the path for performance expectancy and hedonic motivation based on Venkatesh et al. [47] arguments who discussed the influence of financial, intrinsic and functional benefits on users’ behavioural intention. Thus, this study contributed by following the argument of Venkatesh et al [47], and validating the suggestion of Alalwan et al. [43] by providing causal relations between performance expectancy, price value and hedonic motivation, such as investigating the direct impact of performance expectancy on price value concurrently investigating the role of hedonic motivation on both performance expectancy and price value. Furthermore, the current research also augmented the body of existing knowledge by examining the moderating role of performance expectancy and price value. The findings of new relationships which did not exist in earlier literature in the perspective of cryptocurrency adoption enable scholars to have a sound understanding of factors influencing the adoption of a new technology, particularly in the context of Malaysia.

Lastly, unlike all extant literature which investigated the single stage linear and compensatory associations by using structural equational modelling (SEM) [29, 37, 48, 98] it is not believed satisfactory to predict the intricacies relating to complicated decision-making process. Though, to eliminate such limits, researchers [26, 27] have applied ANN as second stage analysis involving of single hidden layer. Huang and Stokes [32] posited that second stage ANN analysis with single hidden layer is a shallow type of ANN.

Consequently, it was suggested to employ deep ANN architecture with more than one hidden layer [34]. Thus, the application of deep ANN architecture with two or more hidden layers will advance the precision of non-linear relationships in the model owing to its deep learning capability [33]. Hence, researchers in this study achieved bridging the present research gap by employing deep learning dual stage approach in order to discover both linear and non-linear compensatory relationships. Moreover, this study further contributes by applying a dual stage deep learning PLS-SEM & ANN analysis. The adoption of the deep learning dual stage approach PLS-SEM & ANN in examining user’s intention to adopt cryptocurrency is believed to be a novel method since previous research on cryptocurrency adoption, i.e. Shoaib et al. [29], was undertaken without exploiting second stage analysis, i.e. deep ANN architecture. The use of artificial intelligence (ANN) and machine learning technique, which is regarded as robust against the noise in the dataset and is capable of learning from the data, enables the researcher to obtain better predictions and to detect non-linear associations that have been ignored in the extant literature.

Additionally, the aforementioned different, fresh, yet useful approach has given robustness to our proposed research model. This dual stage deep learning method is considered vital because users’ behaviour intention to adopt cryptocurrency may not be fully explained by the linear relationships as the decision-making process of humans is already very complex; thus using linear and compensatory associations may oversimplify the human decision-making procedure [101]. Therefore, it can be concluded that with the use of artificial intelligence and expert system of the neural network, the researchers are able to make better predictions and gain better results. The findings of non-linear associations are a breakthrough in the extant literature of cryptocurrency-related literature, particularly in the context of a developing nation like Malaysia, which may serve as an inspiration for scholars to further follow the subject matter in future.

### Practical implications

From a practical perspective, the statistical evidence gained in this study also has significant implications. Results of this study will enable service providers to develop strategies which can encourage the use of cryptocurrency in the daily lives of users by focusing on making the process of using cryptocurrency more useful, easy, enjoyable and trustworthy. The implementation of cryptocurrency technology is not practicable unless customers extensively embrace such novel technologies as a replacement over other channels. In this regard, local financial institutions and regulators may both demonstrate the benefits of adopting cryptocurrency to enhance the adoption rate in country.

Moreover, this empirical study aids in recognising the essential characteristics, consequently, altering the conventional payment method in Malaysia to device appropriate and effective strategies, both at private and government levels to enhance the adoption of such innovative and novel technologies. The results of this study have demonstrated that if users find the technology easy to use and enable users to accomplish the task, the chances for its adoption and subsequent usage becomes higher. Technology-makers need to pay heed to these factors so that adoption of this novel technology can provide widespread benefits, especially to developing economies. At the same time, it is also vital for policymakers to notice the rising importance and growth of this technology, which is frequently mentioned and prominently addressed by both by private (venture capitalists) and public sector (government) [7]. This technology is considered to be a provider of convenient services which are less dependent on third parties, such as commerce, especially pertaining to its low cost [6].

### Limitations and future suggestions

The current has minor limitations. Firstly, the study was cross-sectional, and therefore it is inept at validating changes in human behaviour over time. Thus, for the future, the researcher suggests conducting longitudinal studies to comprehend the progressive changes which can affect a user’s acceptance of cryptocurrency. Second, the study was conducted in Malaysia; therefore, it cannot be generalised to other countries. The researcher suggests that in future, cross-cultural studies must be conducted to validate this model further and to see if this model works in the western context too. Thirdly, future researchers must also examine the post-adoption behaviour of cryptocurrency adopters to uncover the factors which keep stimulating continuous usage. Lastly, while applying the deep neural networks, future scholars must compare the findings of ANN with single layer and more than one layer, so as to reach to a consensus for best architecture.

## Conclusion

Without a doubt, the introduction of the new financial market in the form of digital currencies has transformed the international market space. The aim of this research was to investigate the factors behind the adoption of cryptocurrency by considering personal innovativeness as a moderator between performance expectancy and price value. This study also investigated the moderating role of PI. Findings depict that trust, performance expectancy, price value, effort expectancy and personal innovativeness have a positive influence on user’s behavioural intention to adopt cryptocurrency. Also, the results have also shown support to the new relationship path in comparison to the original model UTAUT2. Furthermore, the researchers found a significantly positive effect of personal innovativeness and price value. These findings open the vistas of knowledge not only theoretically, but also practically, to benefit technology makers and marketers to recognise the crucial elements which may stimulate individuals to adopt cryptocurrency for commercial purposes. Thus, creating an urge to coordinate and agglomerate the industry in a way that fosters trust and increases user confidence.

## Supporting information

S1 File (PDF)

## References

[1] Poongodi T, Sujatha R, Sumathi D, Suresh P, Balamurugan B. Blockchain in social networking. Cryptocurrencies and Blockchain Technology Applications. 2020 6 11:55–76.

[2] Kavanagh D, Ennis PJ. Cryptocurrencies and the emergence of blockocracy. The Information Society. 2020 8 6;36(5):290–300.

[3] Mazambani L. and Mutambara E. (2019). Predicting FinTech innovation adoption in South Africa: the case of cryptocurrency. African Journal of Economic and Management Studies. 2019 10 21.

[4] Winter JS, Davidson E. Big data governance of personal health information and challenges to contextual integrity. The Information Society. 2019 1 1;35(1):36–51.

[5] Al-Amri R, Zakaria NH, Habbal A, Hassan S. Cryptocurrency adoption: current stage, opportunities, and open challenges. International Journal of Advanced Computer Research. 2019 9 1;9(44):293–307.

[6] Omane-Adjepong M, Alagidede IP. High-and low-level chaos in the time and frequency market returns of leading cryptocurrencies and emerging assets. Chaos, Solitons & Fractals. 2020 3 1;132:109563.

[7] Gil-Alana LA, Abakah EJ, Rojo MF. Cryptocurrencies and stock market indices. Are they related?. Research in International Business and Finance. 2020 1 1;51:101063.

[8] Arias-Oliva M, Pelegrín-Borondo J, Matías-Clavero G. Variables influencing cryptocurrency use: a technology acceptance model in Spain. Frontiers in Psychology. 2019 3 ;10:475. 10.3389/fpsyg.2019.00475 30949085 PMC6436067

[9] Hileman G, Rauchs M. Global cryptocurrency benchmarking study. Cambridge Centre for Alternative Finance. 2017 4 7;33:33–113.

[10] Jonker N. What drives the adoption of crypto-payments by online retailers?. Electronic Commerce Research and Applications. 2019 5 1;35:100848.

[11] Seetharaman A, Saravanan AS, Patwa N, Mehta J. Impact of Bitcoin as a world currency. Accounting and Finance Research. 2017 5;6(2):230–46.

[12] Polasik M, Piotrowska AI, Wisniewski TP, Kotkowski R, Lightfoot G. Price fluctuations and the use of bitcoin: An empirical inquiry. International Journal of Electronic Commerce. 2015 9 15;20(1):9–49.

[13] Cong L, Li Y, Wang N. Tokenomics: Dynamic adoption and valuation (Working Paper No. 63). Columbia Business School Research Paper. 2019.

[14] Brick K, Visser M. Risk preferences, technology adoption and insurance uptake: A framed experiment. Journal of Economic Behavior & Organization. 2015 10 1;118:383–96.

[15] Patil P, Tamilmani K, Rana NP, Raghavan V. Understanding consumer adoption of mobile payment in India: Extending Meta-UTAUT model with personal innovativeness, anxiety, trust, and grievance redressal. International Journal of Information Management. 2020 10 1;54:102144.

[16] Kshetri N. Will blockchain emerge as a tool to break the poverty chain in the Global South?. Third World Quarterly. 2017 8 3;38(8):1710–32.

[17] Miraz MH, Ali M. Applications of blockchain technology beyond cryptocurrency. arXiv preprint arXiv:1801.03528. 2018 1 4.

[18] De Filippi P. Bitcoin: a regulatory nightmare to a libertarian dream. Internet Policy Review. 2014 5 14;3(2).

[19] Bitcoin.com. Malaysia Starts Regulating Cryptocurrencies Today. (2019). Retrieved from https://news.bitcoin.com/malaysia-regulating-cryptocurrencies/ Accessed on 1st December, 2019.

[20] Nguyen QK. Blockchain-a financial technology for future sustainable development. In2016 3rd International conference on green technology and sustainable development (GTSD) 2016 Nov 24 (pp. 51–54). IEEE.

[21] Risius M, Spohrer K. A blockchain research framework. Business & Information Systems Engineering. 2017 12;59(6):385–409.

[22] Abbasi G, Su-Yee S, Goh YN. An Integrative Approach for Determining Consumers Mobile Advertising Related Attitudes and Intentions. International Journal of Interactive Mobile Technologies. 2020;14(15), 95–110.

[23] Loh XM, Lee VH, Tan GW, Hew JJ, Ooi KB. Towards a cashless society: the imminent role of wearable technology. Journal of Computer Information Systems. 2019 11 27:1–1.

[24] Glavee-Geo R, Shaikh AA, Karjaluoto H. Mobile banking services adoption in Pakistan: are there gender differences?. International Journal of Bank Marketing. 2017 10 2.

[25] Bol N, Helberger N, Weert JC. Differences in mobile health app use: a source of new digital inequalities?. The Information Society. 2018 5 27;34(3):183–93.

[26] Talukder MS, Sorwar G, Bao Y, Ahmed JU, Palash MA. Predicting antecedents of wearable healthcare technology acceptance by elderly: A combined SEM-Neural Network approach. Technological Forecasting and Social Change. 2020 1 1;150:119793.

[27] Zabukovšek SS, Kalinic Z, Bobek S, Tominc P. SEM–ANN based research of factors’ impact on extended use of ERP systems. Central European Journal of Operations Research. 2019 9;27(3):703–35.

[28] Higueras-Castillo E, Kalinic Z, Marinkovic V, Liébana-Cabanillas FJ. A mixed analysis of perceptions of electric and hybrid vehicles. Energy Policy. 2020 Jan 1;136:111076.

[29] Sohaib O, Hussain W, Asif M, Ahmad M, Mazzara M. A PLS-SEM neural network approach for understanding cryptocurrency adoption. IEEE Access. 2019 Dec 16;8:13138–50.

[30] Alam MZ, Hu W, Kaium MA, Hoque MR, Alam MM. Understanding the determinants of mHealth apps adoption in Bangladesh: A SEM-Neural network approach. Technology in Society. 2020 5 1;61:101255.

[31] Shahzad F, Xiu G, Khan MA, Shahbaz M. Predicting the adoption of a mobile government security response system from the user’s perspective: An application of the artificial neural network approach. Technology in Society. 2020 8 1;62:101278.

[32] Huang W, Stokes JW. MtNet: a multi-task neural network for dynamic malware classification. InInternational conference on detection of intrusions and malware, and vulnerability assessment 2016 7 7 (pp. 399–418). Springer, Cham.

[33] Lee VH, Hew JJ, Leong LY, Tan GW, Ooi KB. Wearable payment: A deep learning-based dual-stage SEM-ANN analysis. Expert Systems with Applications. 2020 11 1;157:113477.

[34] Wang L, Zhang J, Liu P, Choo KK, Huang F. Spectral–spatial multi-feature-based deep learning for hyperspectral remote sensing image classification. Soft Computing. 2017 1 1;21(1):213–21.

[35] Kapoor KK, Dwivedi YK, Williams MD. Innovation adoption attributes: a review and synthesis of research findings. European Journal of Innovation Management. 2014 8 5.

[36] Patel KJ, Patel HJ. Adoption of internet banking services in Gujarat. International Journal of Bank Marketing. 2018 2 5;36(1), 147–169.

[37] Merhi M, Hone K, Tarhini A. A cross-cultural study of the intention to use mobile banking between Lebanese and British consumers: Extending UTAUT2 with security, privacy and trust. Technology in Society. 2019 11 1;59:101151.

[38] Alalwan AA, Dwivedi YK, Rana NP, Algharabat R. Examining factors influencing Jordanian customers’ intentions and adoption of internet banking: Extending UTAUT2 with risk. Journal of Retailing and Consumer Services. 2018 1 1;40:125–38.

[39] Phonthanukitithaworn C, Sellitto C, Fong MW. User intentions to adopt mobile payment services: A study of early adopters in Thailand. Journal of Internet Banking and Commerce. 2015;20(1).

[40] Slade EL, Dwivedi YK, Piercy NC, Williams MD. Modeling consumers’ adoption intentions of remote mobile payments in the United Kingdom: extending UTAUT with innovativeness, risk, and trust. Psychology & Marketing. 2015 8;32(8):860–73.

[41] Oliveira T, Thomas M, Baptista G, Campos F. Mobile payment: Understanding the determinants of customer adoption and intention to recommend the technology. Computers in Human Behavior. 2016 8 1;61:404–14.

[42] Agarwal R, Prasad J. A conceptual and operational definition of personal innovativeness in the domain of information technology. Information systems research. 1998 6;9(2):204–15.

[43] Alalwan AA, Dwivedi YK, Rana NP. Factors influencing adoption of mobile banking by Jordanian bank customers: Extending UTAUT2 with trust. International Journal of Information Management. 2017 6 1;37(3):99–110.

[44] Davis FD. Perceived usefulness, perceived ease of use, and user acceptance of information technology. MIS quarterly. 1989 9 1:319–40.

[45] Beh PK, Ganesan Y, Iranmanesh M, Foroughi B. Using smartwatches for fitness and health monitoring: the UTAUT2 combined with threat appraisal as moderators. Behaviour & Information Technology. 2019 11 2:1–8.

[46] Venkatesh V, Morris MG, Davis GB, Davis FD. User acceptance of information technology: Toward a unified view. MIS quarterly. 2003 9 1:425–78.

[47] Venkatesh V, Thong JY, Xu X. Consumer acceptance and use of information technology: extending the unified theory of acceptance and use of technology. MIS quarterly. 2012 Mar 1:157–78.

[48] Shaw N, Sergueeva K. The non-monetary benefits of mobile commerce: Extending UTAUT2 with perceived value. International Journal of Information Management. 2019 4 1;45:44–55.

[49] Rogers EM. Diffusion of innovations. Simon and Schuster; 2010 7 6. 10.1097/IMI.0b013e3181cf897d 22437269

[50] Ho SH, Ko YY. Effects of self‐service technology on customer value and customer readiness: The case of Internet banking. Internet research. 2008 8 15.

[51] Rodrigues LF, Oliveira A, Costa CJ. Does ease-of-use contributes to the perception of enjoyment? A case of gamification in e-banking. Computers in Human Behavior. 2016 8 1;61:114–26.

[52] Zhou T. Examining users’ switch from online banking to mobile banking. International Journal of Networking and Virtual Organisations. 2018;18(1):51–66.

[53] Ramayah T. Interface characteristics, perceived ease of use and intention to use an online library in Malaysia. Information development. 2006 5;22(2):123–33.

[54] Duarte P, Pinho JC. A mixed methods UTAUT2-based approach to assess mobile health adoption. Journal of Business Research. 2019 9 1;102:140–50.

[55] Lunney A, Cunningham NR, Eastin MS. Wearable fitness technology: A structural investigation into acceptance and perceived fitness outcomes. Computers in Human Behavior. 2016 Dec 1;65:114–20.

[56] Adapa A, Nah FF, Hall RH, Siau K, Smith SN. Factors influencing the adoption of smart wearable devices. International Journal of Human–Computer Interaction. 2018 5 4;34(5):399–409.

[57] Nseke P. How crypto-currency can decrypt the global digital divide: bitcoins a means for African emergence. International Journal of Innovation and Economic Development. 2018;3(6):61–70.

[58] Van der Heijden H., 2004. User acceptance of hedonic information systems. MIS Q. 28 (4), 695–704.

[59] Riffai MM, Grant K, Edgar D. Big TAM in Oman: Exploring the promise of on-line banking, its adoption by customers and the challenges of banking in Oman. International journal of information management. 2012 6 1;32(3):239–50.

[60] Köksal Y, Penez S. An investigation of the important factors influence web trust in online shopping. Journal of Marketing & Management. 2015 5 1;6(1).

[61] Kwateng KO, Atiemo KA, Appiah C. Acceptance and use of mobile banking: an application of UTAUT2. Journal of Enterprise Information Management. 2019 2 11.

[62] Gupta A, Dogra N, George B. What determines tourist adoption of smartphone apps?. Journal of Hospitality and Tourism Technology. 2018 3 12.

[63] Yi MY, Fiedler KD, Park JS. Understanding the role of individual innovativeness in the acceptance of IT‐based innovations: Comparative analyses of models and measures. Decision Sciences. 2006 8;37(3):393–426.

[64] Xu H, Gupta S. The effects of privacy concerns and personal innovativeness on potential and experienced customers’ adoption of location-based services. Electronic Markets. 2009 8 1;19(2–3):137–49.

[65] Farooq MS, Salam M, Jaafar N, Fayolle A, Ayupp K, Radovic-Markovic M, et al. Acceptance and use of lecture capture system (LCS) in executive business studies. Interactive Technology and Smart Education. 2017 11 20.

[66] Dutta DK, Gwebu KL, Wang J. Personal innovativeness in technology, related knowledge and experience, and entrepreneurial intentions in emerging technology industries: a process of causation or effectuation?. International Entrepreneurship and Management Journal. 2015 9;11(3):529–55.

[67] Bhattacherjee A. Social science research: Principles, methods, and practices. 2012.

[68] Sekaran U., & Bougie R. Research for Business–A Skill Building Approach. 2016

[69] Statista (2020). Number of Facebook users in Malaysia from 2017 to 2025. Retrieved from https://www.statista.com/statistics/490484/number-of-malaysia-facebook-users/. Accessed on 05th October 2020.

[70] Kline RB. Assessing statistical aspects of test fairness with structural equation modelling. Educational Research and Evaluation. 2013 4 1;19(2–3):204–22.

[71] Deb M, Lomo-David E. An empirical examination of customers’ adoption of m-banking in India. Marketing Intelligence & Planning. 2014 5 27.

[72] Henseler J, Ringle CM, Sarstedt M. A new criterion for assessing discriminant validity in variance-based structural equation modeling. Journal of the academy of marketing science. 2015 1;43(1):115–35.

[73] Chin WW, Marcolin BL, Newsted PR. A partial least squares latent variable modeling approach for measuring interaction effects: Results from a Monte Carlo simulation study and an electronic-mail emotion/adoption study. Information systems research. 2003 6;14(2):189–217.

[74] Herrero Á, San Martín H. Explaining the adoption of social networks sites for sharing user-generated content: A revision of the UTAUT2. Computers in Human Behavior. 2017 6 1;71:209–17.

[75] Rönkkö M, Evermann J. A critical examination of common beliefs about partial least squares path modeling. Organizational Research Methods. 2013 7;16(3):425–48.

[76] Hair J. F. Jr, Sarstedt M., Hopkins L., & Kuppelwieser V. G. Partial least squares structural equation modeling (PLS-SEM). European business review. 2014; 26(2), 106–121.

[77] Abbasi GA, Jagaveeran M, Goh YN, Tariq B. The impact of type of content use on smartphone addiction and academic performance: Physical activity as moderator. Technology in Society. 2021 2 1;64:101521.

[78] Hamidi H, Chavoshi A. Analysis of the essential factors for the adoption of mobile learning in higher education: A case study of students of the University of Technology. Telematics and Informatics. 2018 7 1;35(4):1053–70.

[79] Ramayah T, Cheah J, Chuah F, Ting H, Memon MA. Partial least squares structural equation modeling (PLS-SEM) using smartPLS 3.0. 2018.

[80] Henseler J, Ringle CM, Sinkovics RR. The use of partial least squares path modeling in international marketing. InNew challenges to international marketing 2009 3 6. Emerald Group Publishing Limited.

[81] Simpson P. K. Artificial neural systems: foundations, paradigms, applications, and implementations. Pergamon. 1990.

[82] Sim JJ, Tan GW, Wong JC, Ooi KB, Hew TS. Understanding and predicting the motivators of mobile music acceptance–a multi-stage MRA-artificial neural network approach. Telematics and Informatics. 2014 11 1;31(4):569–84.

[83] Podsakoff PM, MacKenzie SB, Podsakoff NP. Sources of method bias in social science research and recommendations on how to control it. Annual review of psychology. 2012 1 10;63:539–69. 10.1146/annurev-psych-120710-100452 21838546

[84] Lowry PB, Gaskin J. Partial least squares (PLS) structural equation modeling (SEM) for building and testing behavioral causal theory: When to choose it and how to use it. IEEE transactions on professional communication. 2014 4 22;57(2):123–46.

[85] Bagozzi RP, Dholakia UM, Basuroy S. How effortful decisions get enacted: The motivating role of decision processes, desires, and anticipated emotions. Journal of Behavioral Decision Making. 2003 10;16(4):273–95.

[86] Hair JF, Risher JJ, Sarstedt M, Ringle CM. When to use and how to report the results of PLS-SEM. European business review. 2019 1 14.

[87] Kline R. B. Mean structures and latent growth models. Principles and Practice of Structural Equation Modeling, 4th Edn. The Guildford Press, New York. 2016; 369–393.

[88] Kock N, Lynn G. Lateral collinearity and misleading results in variance-based SEM: An illustration and recommendations. Journal of the Association for information Systems. 2012 9 26;13(7).

[89] Hair JF, Celsi MW, Money AH, Samouel P, Page MJ. Essentials of business research methods: ME Sharpe. Armonk, NY. 2011.

[90] Cohen J. Statistical power analysis for the behavioral sciences. Academic press; 2013 9 3.

[91] Fornell C. & Cha J. Partial least squares, in: Bagozzi R. P.(Ed.) Advanced Methods of Marketing Research Cambridge, MA: Blackwell Pubslishers. 1994.

[92] Shmueli G, Sarstedt M, Hair JF, Cheah JH, Ting H, Vaithilingam S, et al. Predictive model assessment in PLS-SEM: guidelines for using PLSpredict. European Journal of Marketing. 2019 11 11.

[93] Liébana-Cabanillas F, Marinkovic V, de Luna IR, Kalinic Z. Predicting the determinants of mobile payment acceptance: A hybrid SEM-neural network approach. Technological Forecasting and Social Change. 2018 4 1;129:117–30.

[94] Leong LY, Hew TS, Ooi KB, Lee VH, Hew JJ. A hybrid SEM-neural network analysis of social media addiction. Expert Systems with Applications. 2019 11 1;133:296–316.

[95] Läpple D, Van Rensburg T. Adoption of organic farming: Are there differences between early and late adoption?. Ecological economics. 2011 5 15;70(7):1406–14.

[96] MCMC. Interet users survey. (2019). Retrieved from https://www.mcmc.gov.my/skmmgovmy/media/General/pdf/MCMC-Internet-Users-Survey-2017.pdf Accessed 13th December, 2019.

[97] Statista (2019). Share of hand phone users who owned a smartphone in Malaysia as of September 2017, by age group. (2019). Retrieved from https://www.statista.com/statistics/973694/malaysia-smartphone-ownership-by-age/ Accessed on 1st December, 2019.

[98] Sahibzada UF, Cai J, Latif KF, Sahibzada HF. Knowledge management processes, knowledge worker satisfaction, and organizational performance. Aslib Journal of Information Management. 2019 12 17.

[99] Beza E, Reidsma P, Poortvliet PM, Belay MM, Bijen BS, Kooistra L. Exploring farmers’ intentions to adopt mobile Short Message Service (SMS) for citizen science in agriculture. Computers and Electronics in Agriculture. 2018 8 1;151:295–310.

[100] Hossain A, Quaresma R, Rahman H. Investigating factors influencing the physicians’ adoption of electronic health record (EHR) in healthcare system of Bangladesh: An empirical study. International Journal of Information Management. 2019 2 1;44:76–87.

[101] Hew TS, Leong LY, Ooi KB, Chong AY. Predicting drivers of mobile entertainment adoption: a two-stage SEM-artificial-neural-network analysis. Journal of Computer Information Systems. 2016 10 1;56(4):352–70.

