# Supplementary material for: The adoption of cryptocurrency as a disruptive force: Deep learning-based dual stage structural equation modelling and artificial neural network analysis
Source: PLoS One. 2021 Mar 8;16(3):e0247582. doi: 10.1371/journal.pone.0247582 (PMC7939260; doi:10.1371/journal.pone.0247582)
Supplement: S1 File — (PDF) [file pone.0247582.s001.pdf]

Appendix - Online Questionnaires

The adoption of Cryptocurrency as a Disruptive Force: Deep Learning-Based Dual Stage Structural Equation Modelling and Artificial Neural Network Analysis

Dear Respondent,

All information provided in the questionnaire will be strictly treated as confidential and will be only used for research purposes.

Your kind cooperation and assistance in completing the attached questionnaire is greatly appreciated. It would be grateful if you could return the completed questionnaire.

You have any concerns or need for clarifications, please do not hesitate to contact me.

Thank you for your valuable time and cooperation. Your contribution towards this study is highly appreciated.

*Required

1. **Email address** *

____

Section A: Filtering

2. **Nationality** *

This questionnaire is aim for Malaysians nationality only. Thank you!

Mark only one oval.

- ( ) Yes
- ( ) No

3. Age *

This questionnaire is aim for Malaysian adults only. Thank you!

Mark only one oval.

- ⬭ 18 - 25
- ⬭ 26 - 35
- ⬭ 36 - 45
- ⬭ 46 and above

WHAT IS CRYPTOCURRENCY?

- a **VIRTUAL** coinage system that can function much like standard currency (i.e. paper notes and coins) that enabling users to provide **VIRTUAL** payment for goods and services FREE OF A CENTRAL TRUSTED AUTHORITY (such as Bank).
- Some notable cryptocurrencies are Bitcoin, Ethereum, Litecoin and etc.

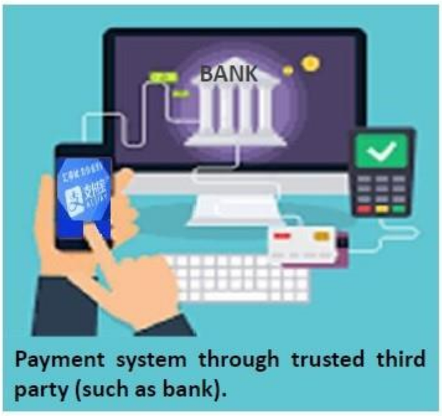

Payment system through trusted third party (such as bank).

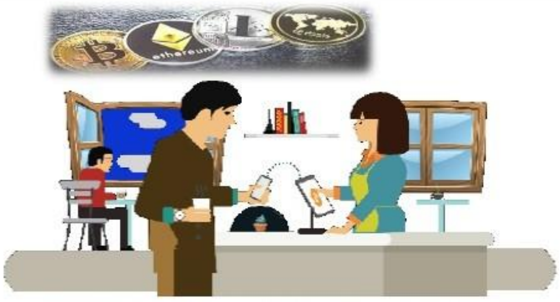

Cryptocurrencies payment system : WITHOUT go through trusted third party (peer-to peer decentralized system).

Section B: Socio-demographic Profile

In the following questions, we would like you to provide some information about yourself. This is just meant for analysis and will keep strictly confidential. Please select for the most appropriate answer that best represent the extent to which you agree with the statements.

4. Residence State*

Mark only one oval.

- ⬭ Penang
- ⬭ Selangor
- ⬭ Johor
- ⬭ Other: ____________________

5. Gender *

Mark only one oval.

- ⬭ Female
- ⬭ Male

6. **Marital Status** *

Mark only one oval.

- Single
- Married
- Other: ____________________

7. **Gross Monthly Income** *

Mark only one oval.

- Below RM 2,000
- RM 2,000 - RM 5,000
- RM 5,001 - RM 10,000
- RM 10,001 and above

8. **Highest Education Level** *

Mark only one oval.

- High School
- Diploma
- Bachelor Degree
- Master Degree
- PhD

Section C: Measurements

This section will assess on the factors affecting the behavioural intention to adopt cryptocurrencies. Please select for the most appropriate answer that best represent the extent to which you agree with the statements.

Performance Expectancy

9. **I find Cryptocurrencies useful in my daily life.** *

Mark only one oval.

	1	2	3	4	5	
Strongly Disagree						Strongly Agree

10. **Using Cryptocurrencies increases my chances of achieving tasks that are important to me.** *

Mark only one oval.

	1	2	3	4	5	
Strongly Disagree						Strongly Agree

11. **Using Cryptocurrencies help me accomplish tasks more quickly.** *
Mark only one oval.

	1	2	3	4	5	
Strongly Disagree	◯	◯	◯	◯	◯	Strongly Agree

12. **Using Cryptocurrencies increase my productivity.** *
Mark only one oval.

	1	2	3	4	5	
Strongly Disagree	◯	◯	◯	◯	◯	Strongly Agree

Effort Expectancy

13. **Learning how to use Cryptocurrencies is easy for me.** *
Mark only one oval.

	1	2	3	4	5	
Strongly Disagree	◯	◯	◯	◯	◯	Strongly Agree

14. **My interaction with Cryptocurrencies is clear and understandable.** *
Mark only one oval.

	1	2	3	4	5	
Strongly Disagree	◯	◯	◯	◯	◯	Strongly Agree

15. **I find Cryptocurrencies easy to use.** *
Mark only one oval.

	1	2	3	4	5	
Strongly Disagree	◯	◯	◯	◯	◯	Strongly Agree

16. **It is easy for me to become skilful at using Cryptocurrencies.** *
Mark only one oval.

	1	2	3	4	5	
Strongly Disagree	◯	◯	◯	◯	◯	Strongly Agree

Social Influence

17. **People who are important to me think that I should use Cryptocurrencies.** *
Mark only one oval.

	1	2	3	4	5	
Strongly Disagree	◯	◯	◯	◯	◯	Strongly Agree

18. **People who influence my behavioural think that I should use Cryptocurrencies.** *
Mark only one oval.

	1	2	3	4	5	
Strongly Disagree	◯	◯	◯	◯	◯	Strongly Agree

19. **People whose opinions that I value prefer that I use Cryptocurrencies.** *
Mark only one oval.

	1	2	3	4	5	
Strongly Disagree	◯	◯	◯	◯	◯	Strongly Agree

Facilitating Conditions

20. **I have the resources necessary to use Cryptocurrencies.** *
Mark only one oval.

	1	2	3	4	5	
Strongly Disagree	◯	◯	◯	◯	◯	Strongly Agree

21. **I have the knowledge necessary to use Cryptocurrencies.** *
Mark only one oval.

	1	2	3	4	5	
Strongly Disagree	◯	◯	◯	◯	◯	Strongly Agree

22. **Cryptocurrencies is compatible with other technologies I use.** *
Mark only one oval.

	1	2	3	4	5	
Strongly Disagree	◯	◯	◯	◯	◯	Strongly Agree

23. **I can get help from others when I have difficulties using Cryptocurrencies.** *
Mark only one oval.

	1	2	3	4	5	
Strongly Disagree	◯	◯	◯	◯	◯	Strongly Agree

Hedonic Motivation

24. Using Cryptocurrencies is fun. *
Mark only one oval.

	1	2	3	4	5	
Strongly Disagree	◯	◯	◯	◯	◯	Strongly Agree

25. Using Cryptocurrencies is enjoyable. *
Mark only one oval.

	1	2	3	4	5	
Strongly Disagree	◯	◯	◯	◯	◯	Strongly Agree

26. Using Cryptocurrencies is very entertaining. *
Mark only one oval.

	1	2	3	4	5	
Strongly Disagree	◯	◯	◯	◯	◯	Strongly Agree

Price Value

27. Cryptocurrencies is reasonably priced. *
Mark only one oval.

	1	2	3	4	5	
Strongly Disagree	◯	◯	◯	◯	◯	Strongly Agree

28. Cryptocurrencies is good value for the money. *
Mark only one oval.

	1	2	3	4	5	
Strongly Disagree	◯	◯	◯	◯	◯	Strongly Agree

29. **At the current price, Cryptocurrencies provides a good value.** *
Mark only one oval.

	1	2	3	4	5	
Strongly Disagree	⬭	⬭	⬭	⬭	⬭	Strongly Agree

Trust

30. **I trust Cryptocurrencies to be reliable.** *
Mark only one oval.

	1	2	3	4	5	
Strongly Disagree	⬭	⬭	⬭	⬭	⬭	Strongly Agree

31. **I trust Cryptocurrencies to be secure.** *
Mark only one oval.

	1	2	3	4	5	
Strongly Disagree	⬭	⬭	⬭	⬭	⬭	Strongly Agree

32. **I believe Cryptocurrencies are trustworthy.** *
Mark only one oval.

	1	2	3	4	5	
Strongly Disagree	⬭	⬭	⬭	⬭	⬭	Strongly Agree

33. **I trust Cryptocurrencies.** *
Mark only one oval.

	1	2	3	4	5	
Strongly Disagree	⬭	⬭	⬭	⬭	⬭	Strongly Agree

Personal Innovativeness

34. **If I hear about a new information technology, I would look for ways to experiment with it.** *
Mark only one oval.

	1	2	3	4	5	
Strongly Disagree	⬭	⬭	⬭	⬭	⬭	Strongly Agree

35. **Among my peers, I am usually the first to try out new information technologies.** *
Mark only one oval.

	1	2	3	4	5	
Strongly Disagree	◯	◯	◯	◯	◯	Strongly Agree

36. In general, I am not hesitant to try out new information technologies. *
Mark only one oval.

	1	2	3	4	5	
Strongly Disagree	◯	◯	◯	◯	◯	Strongly Agree

Behavioural Intention

37. I intend to use Cryptocurrencies in the future. *
Mark only one oval.

	1	2	3	4	5	6	7	
Strongly Disagree	◯	◯	◯	◯	◯	◯	◯	Strongly Agree

38. I will always try to use Cryptocurrencies. *
Mark only one oval.

	1	2	3	4	5	6	7	
Strongly Disagree	◯	◯	◯	◯	◯	◯	◯	Strongly Agree

39. I plan to continue use Cryptocurrencies frequently. *
Mark only one oval.

	1	2	3	4	5	6	7	
Strongly Disagree	◯	◯	◯	◯	◯	◯	◯	Strongly Agree

Powered by
Google Forms
